# Condyloma acuminatum mimicking cervical cancer in a pregnant woman and treatment with cryotherapy: A case report

**DOI:** 10.1097/MD.0000000000032273

**Published:** 2022-12-09

**Authors:** Kai-Chieh Chang, Yen-Chang Chen, Dah-Ching Ding

**Affiliations:** a Department of Obstetrics and Gynecology, Hualien Tzu Chi Hospital, Buddhist Tzu Chi Medical Foundation, and Tzu Chi University, Hualien, Taiwan ROC; b Department of Anatomical Pathology, Hualien Tzu Chi Hospital, Buddhist Tzu Chi Medical Foundation, Hualien, Taiwan ROC; c Department of Pathology, School of Medicine, Tzu Chi University, Hualien, Taiwan ROC; d Institute of Medical Sciences, Tzu Chi University, Hualien, Taiwan ROC.

**Keywords:** cervical cancer, condyloma, cryotherapy, pregnancy

## Abstract

Condyloma acuminata and anogenital warts are protruding papillomatous lesions caused by human papillomavirus. In pregnant women, condyloma acuminata over the cervical region may grow rapidly, mimicking cervical cancer.

**Patient concerns:**

A pregnant woman at 14 weeks of gestation with condyloma acuminatum mimicking cervical cancer was referred to our hospital for further management.

**Diagnosis:**

Condyloma acuminata.

**Interventions:**

Tumor biopsy was performed twice, and the pathology confirmed condyloma acuminatum. Immunohistochemistry revealed focal positivity for p16 and Ki-67. Cryotherapy was performed and regular follow-up was performed at 2-week intervals. A small residual condyloma acuminata was found and treated with cryotherapy.

**Outcome:**

During the follow-up period, no recurrence of condyloma acuminata was noted. She delivered a baby at 37 weeks of gestation via cesarean section, without complications.

**Lessons:**

Condyloma acuminata of the cervix may grow faster during pregnancy, mimicking cervical cancer. Multiple factors must be considered when treating condyloma acuminata during pregnancy. Cryotherapy is proposed as a 1^st^-line treatment in all trimesters because of its safety, convenience, and cost-effectiveness. Serial follow-up at 2-week intervals to observe post-cryotherapy conditions is recommended.

## 1. Introduction

Condyloma acuminata is an anogenital wart caused by human papillomavirus (HPV) infection.^[1]^ HPV infection is mainly transmitted through sexual behavior. The development of condyloma acuminata can be affected by lifestyle, age, and sexual activity.

More than 100 HPV serotypes have been identified to date. Low-risk HPV serotypes 6 and 11 are the most common types that cause condyloma acuminata.^[1]^ High-risk HPV (types 16 and 18) can cause cervical cancer in women.^[2]^

The prevalence of HPV infection ranges from 9% to 13% globally. It is also the most common sexually transmitted infection worldwide.^[3]^ Adults aged between 20 and 39 years of age are mostly infected.^[4]^ The risk factors for HPV infection include multiple sexual partners, a history of gonorrhea or chlamydia infections, human immunodeficiency virus infection, and smoking.^[5]^ The incidence of anogenital warts is 1.1 to 1.2 per 1000 person-years.^[6]^

Condyloma acuminata may be asymptomatic or present with symptoms such as itching, pain, and vaginal bleeding.^[7]^ Condyloma acuminata may be discovered for the 1^st^ time during regular perinatal examinations during pregnancy. Pregnant women with condyloma acuminata have a higher chance of vertical mother-to-child transmission.^[8]^ Therefore, it is important to treat condyloma acuminata in pregnant women to decrease the possibility of vertical transmission.^[8]^

Condyloma acuminata in pregnant women tends to proliferate significantly, possibly due to physiological changes in the external genitalia and the partially immunocompromised status of pregnancy, which allows for the rapid replication of HPV.^[9]^ Owing to the downregulation of cell-mediated immunity in pregnancy, pregnant women are prone to have large condyloma acuminata lesions.^[9]^ Apart from the symptoms caused by condyloma acuminata, obstetric complications may include hemorrhage-related impaired wound repair and increased neonatal HPV infection.^[9]^

Multiple treatment options are available for condyloma acuminata. Topical agents, surgical excision, electrocauterization, and cryotherapy are treatment options for condyloma.^[8]^ Cryotherapy is inexpensive, less painful, and safe; thus, it is recommended as 1^st^-line therapy in pregnancy.^[10,11]^

We report the case of a pregnant woman with cervical condyloma mimicking cervical cancer, which was discovered at 14 weeks of gestation and treated with cryotherapy. The baby was delivered via cesarean section because of a previous surgical scar.

## 2. Case report

A 34-years-old pregnant woman presented to our outpatient clinic at 14 weeks of gestation with a chief complaint of a small amount of vaginal bleeding for 2 weeks. The patient was admitted to another hospital where cervical malignancy was suspected, and further evaluation at our medical center was suggested.

Upon examination, she denied having trauma or engaging in recent sexual activity. She denied any known systemic disease, uterine or vaginal structural abnormalities, or medication use. She denied alcohol, betel nut, and cigarette consumption. She was a social worker in a nearby town and denied travel or contact history of respiratory disease. She had had a pregnancy 3 years before this evaluation, which was accompanied with uncomplicated hypertension. The previous delivery was performed at 37 weeks at a hospital due to a premature membrane rupture noted during a routine examination. The planned normal spontaneous delivery was switched to cesarean section because of prolonged labor and breech presentation.

**Figure 1.**
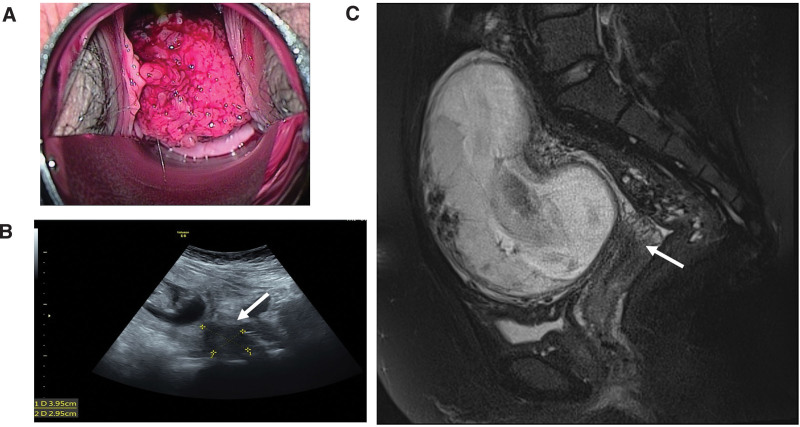
Gross image, ultrasound, and magnetic resonance image (MRI) of the condyloma acuminata. (A) Gross image of the condyloma acuminata. The tumor was 3.5 cm in diameter at the cervical region. (B) Ultrasound of the cervical condyloma acuminata (hypoechoic lesion, arrow). (C) T2 MRI of the cervical condyloma acuminata (arrow). MRI = magnetic resonance image.

Vaginal examination showed a protruding cauliflower-like cervical tumor measuring 2.5 centimeters, with easy contact bleeding (Fig. 1A). Pelvic ultrasound showed a 2.5-centimeter cervical hypoechoic mass (Fig. 1B). She reported normal annual pap smear results in the previous 2 years and denied having multiple sexual partners. A colposcopic biopsy was performed, and pathology showed possible squamous cell carcinoma (SCC); however, further confirmation was required. Pelvic magnetic resonance imaging was performed 2 days later, but the lesion was too subtle to be defined (Fig. 1C). No apparent lymphadenopathy or metastasis in the pelvic region was observed on imaging. The levels of tumor markers [carcinoembryonic antigen, SCC, CA125, and CA19-9] were all within normal limits.

**Figure 2.**
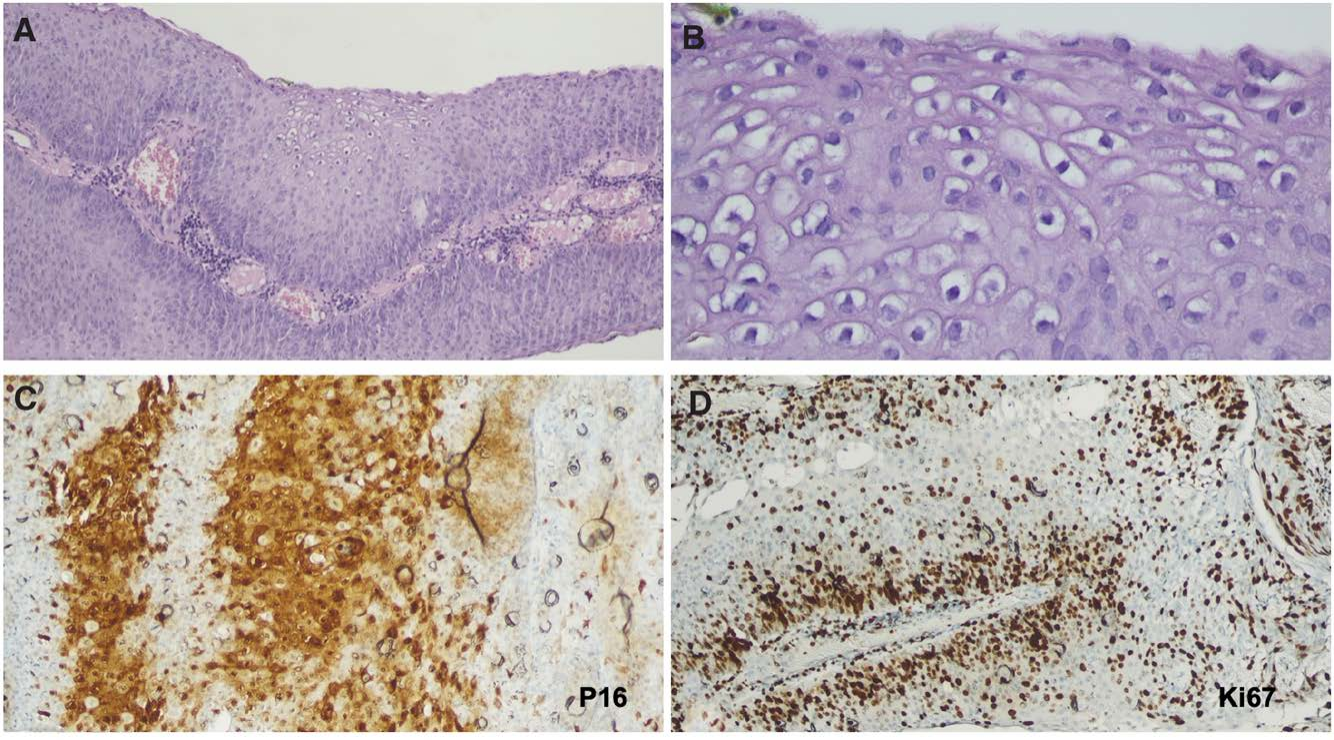
Histopathology and immunohistochemistry of the condyloma acuminata. (A) H&E staining of the condyloma (50×). (B) Magnification of (A) (200×). (C) Immunohistochemistry of p16 (brown color, 200×). (D) Immunohistochemistry of Ki67 (brown color, 100×).

Because of the suspected SCC of the cervix, we performed a repeat cervical biopsy. Pathological examination showed condyloma acuminatum (Fig. 2A and B). Immunohistochemistry revealed p16 focal positivity (30%) (Fig. 2C) and mild-to-moderately increased Ki-67 expression (Fig. 2D). Consequently, she underwent cryotherapy to eradicate the lesion at 16 weeks of gestation. She was followed up at our outpatient clinic every 2 weeks. A small condyloma lesion was noted 1 month later, and she underwent a second cryotherapy. The following course of the pregnancy was uneventful, except for gestational diabetes mellitus, and diet control was suggested. She underwent cesarean section at 37 weeks of gestation, and the baby was delivered uneventfully.

## 3. Discussion

Various clinical conditions and management strategies for condylomas during pregnancy have been reported in the literature. Condyloma may be large and undiagnosed until the patient presents for delivery. The neonate may be delivered through a normal vaginal delivery or cesarean section if insufficient vaginal stretching with possible obstruction is suspected.^[8]^ Surgical excision of large lesions before delivery is seldom considered reasonable management because the fetus may be affected during anesthesia.^[12,13]^

In the present case, the patient 1^st^ visited another hospital, where the lesion was highly likely to be malignant. The lesion showed risky features, including rapid outgrowth and contact bleeding, during her 1^st^ visit to our outpatient clinic. A diagnosis of cervical SCC was considered until the pathology report confirmed that the lesion was a condyloma acuminata. To the best of our knowledge, this is the 1^st^ case report of a condyloma mimicking cancer.

The treatment options for condyloma acuminatum can be divided into patient-applied and provider-applied methods.^[14]^ The former indicates the use of topical imiquimod,^[15]^ and the latter mainly includes laser therapy, cryotherapy, photodynamic therapy, and local hyperthermia.^[16]^ Cryotherapy is recommended as the 1^st^-line therapy because it does not require anesthesia; thus, management in an outpatient setting is possible. Moreover, the risks of local complications and danger to the fetus are low.^[11]^ Laser therapy is recommended as second-line therapy because all lesions can be treated in 1 session, especially extensive lesions.^[8]^

Vertical mother-to-child transmission of HPV can occur in children born via a cesarean section.^[17]^ If no other indications for cesarean delivery are present, prophylactic cesarean section may have limited benefits. However, treating maternal condyloma lesions is important to decrease the overall HPV viral load.^[8]^

p16 and Ki-67 double immunostaining is used to detect high-grade squamous intraepithelial lesions (SIL).^[18]^ The p16 and Ki-67 staining intensities gradually increase as lesions progress from low-grade to high-grade SIL.^[19]^ Diffuse staining for p16 is a marker of high-grade SIL and is not associated with HPV infections.^[20]^ p16 and Ki-67 positivity is associated with histologic severity.^[21]^ Our case showed focal positivity for p16 and Ki-67, and no SIL was found.

A limitation of this report was that it included only 1 case, which may not be representative of other cases. Our case report indicates that condyloma can mimic cervical cancer, but the possibility of cervical cancer even in the presence of condyloma should still be considered.

## 4. Conclusion

Our case showed a large cervical condyloma that mimicked cervical cancer. After pathological confirmation, the condyloma was diagnosed and treated with cryotherapy. Finally, the patient delivered a healthy baby. Our case suggests that pathological confirmation is essential for a condyloma diagnosis. A reasonable treatment plan can be determined if the correct diagnosis is made. We chose cryotherapy for condyloma owing to its convenience and the non-requirement for anesthesia. Serial follow-up after cryotherapy is important for determining treatment efficacy and the need for further treatment sessions. In our case, follow-up every 2 weeks was arranged to monitor for recurrence. The second cryotherapy session was conducted because of a small lesion during regular follow-up. The delivery was through cesarean section due to a previous scar; however, if no other factors exist, vaginal delivery may also be reasonable in a completely treated case of condyloma.

## Author contributions

**Conceptualization:** Dah-Ching Ding.

**Data curation:** Kai-Chieh Chang, Yen-Chang Chen, Dah-Ching Ding.

**Formal analysis:** Kai-Chieh Chang, Dah-Ching Ding.

**Investigation:** Yen-Chang Chen.

**Supervision:** Dah-Ching Ding.

**Writing – original draft:** Kai-Chieh Chang, Dah-Ching Ding.

**Writing - review & editing:** Dah-Ching Ding.
